# Effect of different anaesthetic techniques on the prognosis of patients with colorectal cancer after resection: a systematic review and meta-analysis

**DOI:** 10.3389/fonc.2024.1397197

**Published:** 2024-04-15

**Authors:** Shijun Xia, Yuwen Zhu, Wenjiang Wu, Yue Li, Linchong Yu

**Affiliations:** Shenzhen Hospital of Guangzhou University of Chinese Medicine, Shenzhen, China

**Keywords:** colorectal cancer, inhalation anaesthesia, recurrence, survival, total intravenous anaesthesia

## Abstract

**Background:**

The effect of total intravenous anaesthesia (TIVA) and inhalation anaesthesia (IA) on the prognosis of patients with colorectal cancer after resection is controversial. This study aimed to explore the effects of different anaesthesia methods on the postoperative prognosis of colorectal cancer.

**Methods:**

PubMed, Embase and Cochrane Library databases were searched for relevant literature from each database’s inception until 18 November 2023. The literature topic was to compare the effects of TIVA and IA on the prognosis of patients undergoing colorectal cancer resection.

**Results:**

Six studies were selected for meta-analysis. The studies involved 111043 patients, with a trial size of 1001–88184 people. A statistically significant difference was observed in the overall survival (OS) between colorectal cancer patients administered TIVA and IA (hazard ratio [HR], 0.83; 95% confidence interval [CI], 0.70–0.99), but none in recurrence-free survival (RFS) (HR, 0.99; 95% CI, 0.90–1.08). In the subgroup analysis of OS, no statistically significant difference was observed between colorectal cancer patients administered TIVA and IA in Asia (HR, 0.77; 95% CI, 0.57–1.05), and not in Europe (HR, 0.99; 95% CI, 0.93–1.06). Regarding tumour location, no significant association was found between TIVA and IA in the colon, rectum and colorectum ((HR, 0.70; 95% CI, 0.38–1.28), (HR, 0.95; 95% CI, 0.83–1.08) and (HR, 0.99; 95% CI, 0.93–1.06), respectively).

**Conclusion:**

OS differed significantly between patients administered TIVA and IA when undergoing colorectal cancer resection, but no difference was observed in RFS. The prognostic effects of TIVA and IA differed.

**Systematic review registration:**

https://www.crd.york.ac.uk/prospero/display_record.php?ID=CRD42023453185, identifier CRD42023453185.

## Introduction

Between 2008 and 2018, the number of patients with cancer increased by >25% globally (1, 2), and excisional surgery remains one of the main treatments for solid organ tumours in cancer patients (3). Particularly, colorectal cancer is the fourth deadliest cancer worldwide, with approximately 900000 cases of mortality yearly (4). Surgery is the cornerstone of many treatment options for colorectal cancer. Although it is usually aimed at healing, the removal of tumours is also a risk factor for metastasis. Tumour cells can enter the bloodstream before, during or after surgery, leading to distant organ metastasis (5). The mechanism of metastasis includes carcinomas escaping the immune system, proliferating and invading tissues. Surgery creates a tumorigenic physiological environment that may directly or indirectly affect tumour cell survival.

Multiple perioperative factors collectively contribute to a relatively immunosuppressive state, including surgical stress response and surgical inflammatory response, as well as the direct effects of aneasthetics, opioids and other perioperative drugs. Research has shown that volatile anaesthetics used in inhalation anaesthesia (IA) promote tumour metastasis, which may include direct promotion of carcinoma survival, inhibition of immune cell function and tumour cell-killing function (6–9). Propofol used in total intravenous anaesthesia (TIVA) is the most commonly used intravenous inducer, and some preclinical evidence suggests that it may have anti-tumour effects. Propofol exerts anti-tumour effects by directly regulating key ribonucleic acid pathways and signal transduction in carcinomas (10). It also has anti-inflammatory and anti-oxidant effects (11–16), preventing immune suppression during the perioperative period.

The impact of TIVA and IA on the prognosis of patients with colorectal cancer has always been controversial. Previous research results showed inconsistent trends. A retrospective analysis showed that volatile anaesthetics slightly increased the cancer recurrence rate in patients undergoing colorectal cancer surgery compared with TIVA using propofol (17). Another study (18) showed that there was no difference in overall survival (OS) or recurrence-free survival (RFS) between the two anaesthesia methods for colorectal cancer.

Based on the above controversy, this study aimed to explore the impact of TIVA and IA on the prognosis of patients with colorectal cancer after resection through meta-analysis.

## Methods

### Protocol and guidance

This study was conducted following the Preferred Reporting Items for Systematic Reviews and Meta-analyses reporting guidelines (19). This study did not require ethical approval or informed consent. The protocol for this review has been registered with PROSPERO (CRD42023453185).

### Search strategy

We searched PubMed, EMBASE and the Cochrane Library electronic databases for research written in English from its establishment until 18 November 2023, with keywords including (‘Colorectal Cancer’, ‘colon’ or ‘rectal’), (‘analgesia’, ‘Anesthesia’, ‘Inhalation’ or ‘intravenous’) and (‘Desflurane’, ‘Propofol’, ‘dexmedetomidine’ or ‘Sevoflurane’). Other studies were searched for by reviewing reference lists and qualified publications of potential qualified studies. All searches were conducted independently by two authors, and differences were discussed after the search process.

### Inclusion and exclusion criteria

If the retrieved studies (1) were cohort studies, (2) investigated patients with colorectal cancer, (3) compared clinical studies on long-term all-cause mortality and recurrence after TIVA or IA and (4) provided hazard ratios (HRs) or risk ratios and their 95% confidence intervals (CIs), they were eligible for qualitative and quantitative analyses.

If study participants (1) had malignant tumours other than colorectal cancer and (2) lacked measurements of cancer recurrence or mortality, the study was excluded.

### Data extraction and quality assessment

Two reviewers independently extracted data from the included studies. This review introduced the following details: the name of the first author, year of publication, country, number of participants, tumour location, research type, intervention measures and main research indicators.

The quality of all selected studies was checked according to the Newcastle–Ottawa cohort study quality assessment scale (20). This semi-quantitative scale uses a star rating system to evaluate the quality of eight items in three fields: selection (four items, one star each), comparability (one item, up to two stars) and exposure (three items, one star each). In this meta-analysis, we classified quality as good (≥7 stars), average (4–6 stars) or poor (<4 stars). Differences between the two reviewers were resolved through discussion with the third reviewer.

### Data analysis

Based on the effects of TIVA and IA, the results show RFS and OS in patients with cancer. This method is based on the HR obtained from each study with a 95% CI. If HR was unreported, the odds ratio was considered equal to HR. The study selected TIVA rather than IA. If the data included in the study comprised IA rather than TIVA, then it was adjusted (calculate derivative). When there are multiple sets of useful data in the same study, only data from propensity score matching is selected for analysis. Subgroup analysis was performed based on region (Asia and Europe) and tumour location (colon, rectum and colorectum).

### Statistical analysis

Review manager, version 5.4 (Nordic Cochrane Center, Cochrane Collaboration, London, UK) was used for data analysis. The HR was used to measure effectiveness at a 95% CIs. I^2^ values were used to describe heterogeneity and were categorised into four levels: no heterogeneity (I^2^ < 25%), low heterogeneity (25% ≤ I^2^ < 50%), moderate heterogeneity (50% ≤ I^2^ < 75%) and high heterogeneity (I^2^ ≥ 75%). When the I^2^ value was <50%, a fixed model effect was used, whereas when it was >50%, a random model effect was used.

## Results

### Eligible studies and study characteristics

**Figure 1:**
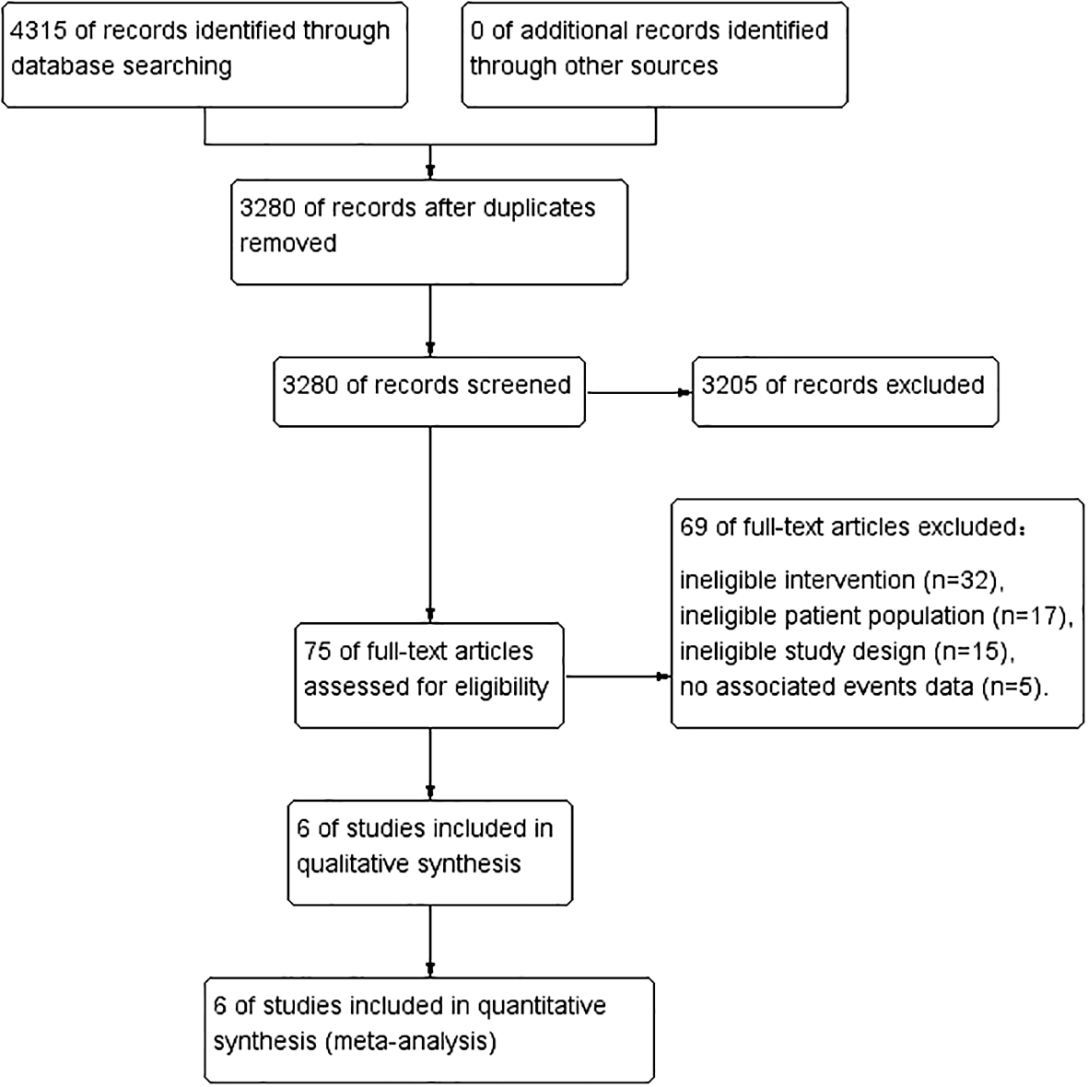
PRISMA flow diagram of study selection.

After identifying 4315 references, 1035 duplicate publications and 3205 irrelevant studies were excluded, leaving 75 potentially eligible studies (**Figure 1**). Finally, six cohort (17, 18, 21–24) studies conducted between 2014 and 2022 were included in the meta-analysis.

**Table 1** lists the general characteristics of the included studies. A total of 111043 patients with cancer participated in the study, with trial sizes of 1001–88184 people. The six studies were retrospective studies using propensity matching scores. The main outcome measures are OS and RFS. Among these studies, two were from Europe and four were from Asia. According to the quality evaluation criteria, all six studies were rated as good quality.

**Table 1:** Characteristics of the included trials.

First Author (Publication year)	Country	Number of participants	Cancer type	Study design	Interventions	Outcomes	Quality assessment
Enlund (2014) (21)	Sweden	1001	Colon, rectal	Retrospective; propensity score matching	Sevoflurane VS Propofol	OS	8
Wu (2018) (22)	China	1158	colon	Retrospective; propensity score matching	Propofol VS Desflurane	OS	7
Makito (2020) (23)	Japan	88184	Colon, rectal	Retrospective; propensity score matching	Desflurane, sevoflurane, or isoflurane with/without nitrous oxide VS Propofol	OS, RFS	9
Hasselager (2021) (17)	Denmark	8694	colorectal	Retrospective; propensity score matching	Sevoflurane VS Propofol	OS, RFS	8
Lee (2022) (18)	Korea	2127	colorectal	Retrospective; propensity score matching	Propofol VS Sevoflurane	OS, RFS	8
Yoon (2022) (24)	Korea	9879	colorectal	Retrospective; propensity score matching	Propofol VS Sevoflurane, desflurane, isoflurane, or enflurane	OS	9

### Recurrence-free survival

**Figure 2:**
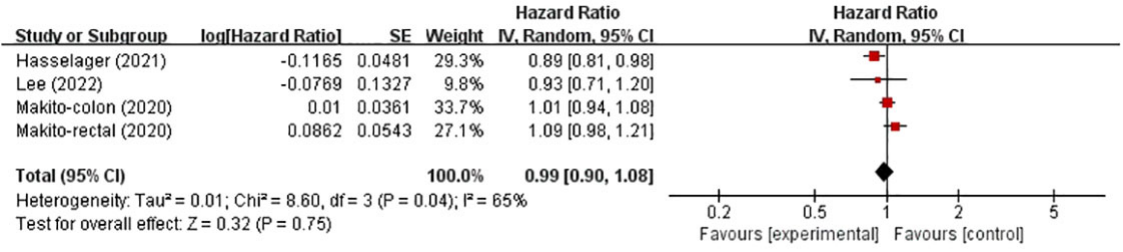
TIVA and IA on RFS of colorectal cancer.

Three studies investigated the effects of TIVA and IA on the RFS rate of colorectal cancer (**Figure 2**). The total sample size was 99005 patients. Compared with IA, the use of TIVA was not associated with an improved RFS rate in colorectal cancer (HR, 0.99; 95% CI, 0.90–1.08; *p* = 0.75).

### Overall survival

**Figure 3:**
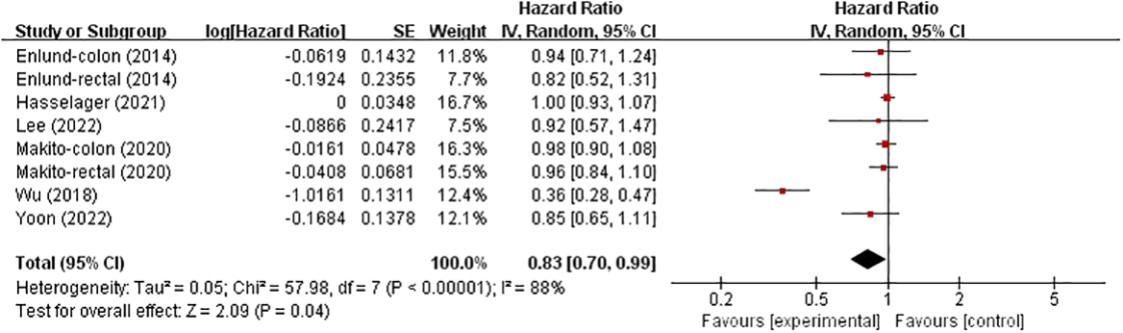
TIVA and IA on OS of colorectal cancer.

six studies investigated the effects of TIVA and IA on OS in colorectal cancer patients (**Figure 3**), involving 111043 patients. Compared with IA, TIVA improved OS (HR, 0.83; 95% CI, 0.70–0.99; *p* = 0.04).

**Figure 4:**
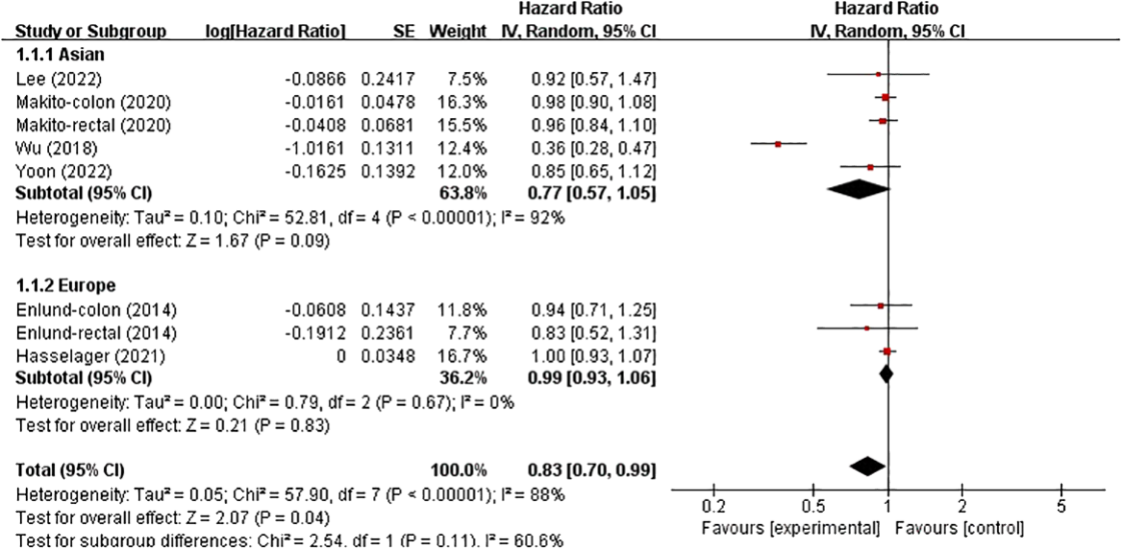
Subgroup analysis based on country with OS.

**Figure 5:**
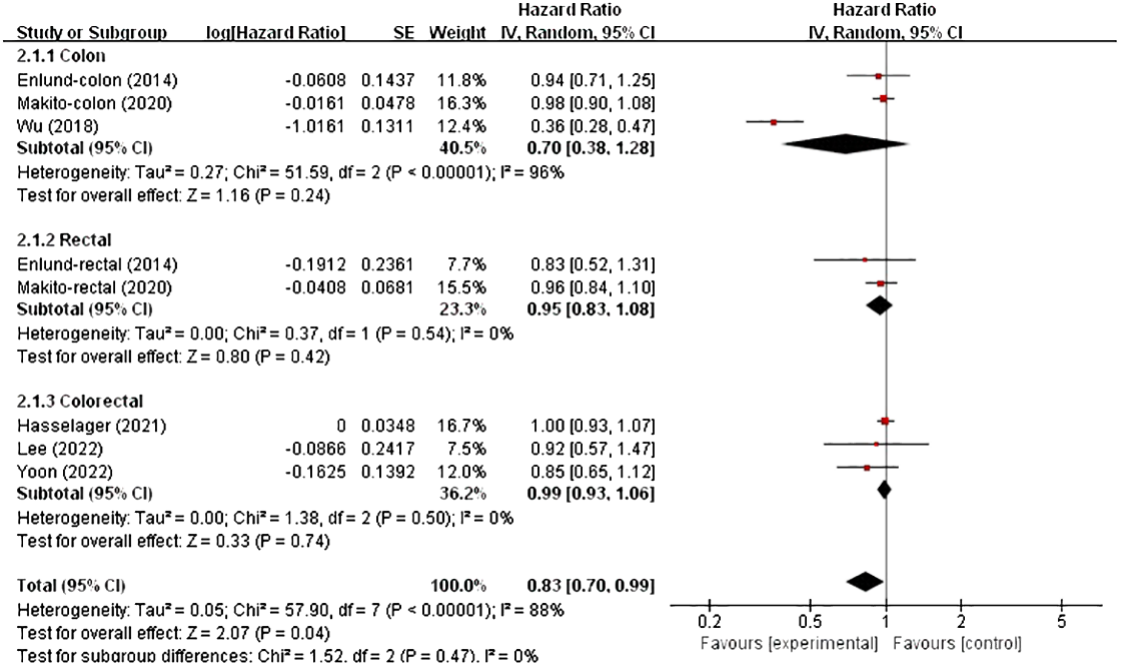
Subgroup analysis based on cancer location with OS.

In these analyses, two studies analysed the colon and rectum, one analysed the colon and the remaining three analysed the colorectum. Subgroup analysis was conducted based on country and cancer location. The results showed no significant correlation between patients from Asia (HR, 0.77; 95% CI, 0.57–1.05; *p* = 0.09), and not between patients from Europe (HR, 0.99; 95% CI, 0.93–1.06; *p* = 0.83) (**Figure 4**). However, in the subgroup analysis of tumour location, no significant associations were found in either the colon, rectum or colorectal tumours (HR, 0.70; 95% CI, 0.38–1.28), (HR, 0.95; 95% CI, 0.83–1.08) and (HR, 0.99; 95% CI, 0.93–1.06) (**Figure 5**).

### Publication bias

In accordance with the criteria in the Cochrane Handbook for systematic reviews of interventions, publication bias was not analysed because none of the groups comprised >10 studies.

## Discussion

Our meta-analysis included six retrospective studies for comparing the effects of TIVA and IA on postoperative prognosis after colorectal cancer resection. The data results processed using propensity score matching reduced the impact of selection bias; therefore, conducting a meta-analysis on these data yielded more consistent and less heterogeneous results. We found a statistically significant difference in OS between TIVA and IA for patients with colorectal cancer, but none in RFS. We conducted a subgroup analysis on OS and found no statistically significant difference between TIVA and IA in patients with colorectal cancer in Asia, and not in Europe. Regarding tumour location, no significant association was found between TIVA and IA in colon, rectum or colorectal cancer.

Propofol is the most commonly used intravenous inducer for anaesthesia maintenance. Some preclinical evidence suggests that it may have anti-tumour effects. Laboratory research has shown that propofol exerts anti-tumour effects by directly regulating key ribonucleic acid pathways and signal transduction in carcinomas (10). It also has anti-inflammatory and anti-oxidant effects, preventing immune suppression during the perioperative period. *In vitro* studies have confirmed that propofol has multiple anti-tumour effects in different cancer cell lines. In gastric cancer cell lines, it inhibits cell proliferation, invasion and migration (25). In non-small cell lung cancer (NSCLC), propofol interferes with HIF1A upregulation, thereby reducing carcinoma migration and invasion (26). In a study of breast cancer cell lines, propofol reduced the expression of neuroepithelial transformation gene 1, which promotes adenocarcinoma migration *in vitro* (27).

Laboratory studies have shown that the mechanisms by which volatile anaesthetics promote tumour metastasis may include the direct promotion of carcinoma survival and inhibition of immune cell and tumour cell-killing functions. However, the molecular mechanism remains unclear, and the evidence for different inhaled drugs and different cancer cell lines is contradictory. Volatile anaesthetics also have pro-inflammatory effects (28). They may upregulate hypoxia-inducible factor (HIF) and protect carcinomas during the perioperative period (29).

The results of clinical research comparing intravenous and inhaled drugs are inconsistent. Regarding the survival rate, a meta-analysis in 2019 included 6 studies, with >7800 patients with breast cancer, oesophageal cancer or NSCLC undergoing surgery. The results revealed that the RFS of TIVA users was higher than that of IA users (summary HR 0.78, 95% CI 0.65–0.94) (30). Regarding circulating tumour cells, a randomised trial included 210 patients undergoing breast cancer surgery. The results showed that the number of circulating tumour cells after surgery was similar among patients treated with sevoflurane and propofol (31). Regarding immune cells, a randomised trial found a similar proportion of NK cells, helper T cells and cytotoxic T cells in postoperative circulation among 153 patients who underwent colorectal cancer resection under sevoflurane- and propofol-induced anaesthesia (32). Regarding tumour regulatory factors, *in vivo* studies have not clarified the effects of intravenous and inhaled drugs on these factors. A small study evaluated the expression of oncogenes in patients undergoing head and neck cancer resection and found a significant increase in HIF1A expression among users of volatile anaesthetics (33).

Regardless of the exact mechanism, the choice of TIVA or VA is a potential modifiable factor in the management of colorectal cancer, and our meta-analysis results indicate that TIVA is associated with lower postoperative mortality. Further prospective clinical trials are required to elucidate the role of anaesthetics in cancer prognosis.

In the assessment of bias risk, we noticed the control of confounding factors with the most prominent bias risk. Many studies have not fully considered confounding factors such as patient comorbidities or tumour grading. For any group wishing to conduct further research on this topic, these issues need to be considered. Furthermore, most studies are retrospective and lack prospective randomised controlled trials.

Finally, although our meta-analysis established a possible association, it inferred no causal relationship nor explained potential mechanisms. We believe that further prospective clinical trials are required to elucidate the molecular mechanisms underlying the role of anaesthetics in cancer prognosis.

In conclusion, we conducted a meta-analysis using six studies, which included 111043 patients, and the results showed an association between TIVA and postoperative mortality in cancer surgery, but its impact on RFS remains inconclusive.

## Data availability statement

The original contributions presented in the study are included in the article/supplementary material. Further inquiries can be directed to the corresponding author.

## Author contributions

SX: Conceptualization, Data curation, Formal analysis, Software, Writing – original draft, Writing – review & editing. YZ: Data curation, Formal analysis, Software, Writing – original draft. WW: Conceptualization, Funding acquisition, Resources, Writing – original draft, Writing – review & editing. YL: Data curation, Formal analysis, Writing – original draft. LY: Data curation, Software, Writing – original draft.
